# Soundscape Optimization in Nursing Homes Through Raising Awareness in Nursing Staff With MoSART+

**DOI:** 10.3389/fpsyg.2022.871647

**Published:** 2022-06-01

**Authors:** Janouk Kosters, Sarah I. M. Janus, Kirsten A. Van Den Bosch, Sytse Zuidema, Hendrika J. Luijendijk, Tjeerd C. Andringa

**Affiliations:** ^1^Department of General Practice and Elderly Care, University Medical Center Groningen, Groningen, Netherlands; ^2^Faculty of Behavioural and Social Sciences, University of Groningen, Groningen, Netherlands; ^3^SoundAppraisal BV, Groningen, Netherlands

**Keywords:** soundscape, nursing home, sound intervention, dementia, trial

## Abstract

**Introduction:**

Soundscapes in nursing homes are often suboptimal for residents. This can cause them feeling anxious and unsafe, or develop behavioral and psychological problems. Residents with dementia cannot adapt nursing home environments to their needs due to cognitive and physical limitations. It is up to the staff of psycho-geriatric wards to improve the soundscape. We evaluated the effect of the sound awareness intervention Mobile Soundscape Appraisal and Recording Technology (MoSART+) on soundscapes in nursing homes.

**Methods:**

The MoSART+ intervention was carried out in four nursing homes and took three months in each home. The MoSART+ intervention involved training ambassadors, assessing the soundscape with the MoSART application by the nursing staff to raise their sound awareness on random time points, discussing the measurements, and implementing micro-interventions. Soundscapes were assessed from 0 to 100 on four attributes: pleasantness, eventfulness, complexity, and range of affordances. Based on these scores, soundscapes were classified in four dimensions: calm, lively, boring, and chaotic. Nursing staff graded the environment on a scale of 0 to 10. T-test and Z-tests were used to analyze data.

**Results:**

Staff recorded 1882 measurements with the MoSART app. “People,” “music, TV, and radio,” and “machines and appliances” were the most prevalent sound sources before and after the implementation of micro-interventions. Post-implementation of micro-interventions, soundscapes were pleasant (median 69.0; IQR 54.0–81.0), of low complexity (33.0; 18.0–47.0), uneventful (27.0; 14.0–46.5), and gave moderate affordances (50.0; 35.0–67.0). Changes in attributes were statistically significant (*p* < 0.01). The proportion of the dimension calm increased (z = 12.7, *p* < 0.01), the proportion of chaotic decreased (z = 15.0, *p* < 0.01), and the proportion of lively decreased not statically significant (z = 0.68, *p* = 0.79). The proportion of boring was unchanged. The proportion of grades ≥6 increased after implementation of the micro-interventions (z = 15.3, *p* < 0.01). The micro-interventions focused on removing or reducing disturbing sounds and were unique for each nursing home.

**Discussion:**

The MoSART+ intervention resulted in a statistically significantly improvement of soundscapes, and more favorable evaluations of the nursing staff. The intervention empowered staff to adapt soundscapes. Nursing homes can improve soundscapes by raising sound awareness among staff.

**Trial Registration:**

Netherlands National Trial Register (NL6831).

## Introduction

Soundscapes are auditory environments as perceived by an individual or society, in a specific context (International Organisation for Standardisation, 2014). Soundscapes influence the well-being of humans through their continuous presence and interaction. Annoying sounds are known to disturb relaxation, and sleep, and cause stress (Babisch, 2002). Humans differ in their sensitivity to noise and their need for quietness (Booi and van den Berg, 2012), which also influences the impact of sound on the health of the individual. Hence, what is perceived as noise or unwanted sound depends on one's current needs, goals, and activities. Also, people's needs, goals and activities change according to different environments (Andringa and Lanser, 2013). This article underlines the importance of soundscapes in nursing homes, which are the homes of the residents but the workplaces of the staff.

Soundscapes in nursing homes are often suboptimal for the residents, because they are full of unexpected, repetitive, loud or droning noises produced by staff, household appliances, and other residents (Schnelle et al., 1993). Residents may perceive the auditory environment as unpleasant, disturbing, and unsafe (van den Bosch et al., 2016). A few studies have investigated how soundscapes influence residents with dementia. They showed that detrimental auditory environments had a negative impact on sleep, and could also promote behavioral problems such as agitation, apathy, and wandering (Schnelle et al., 1998; Garre-Olmo et al., 2012; Joosse, 2012; Van Vracem et al., 2016). People with dementia are highly sensitive to auditory environments, due to diminished cognitive functions and often limited sensory abilities (van Hoof et al., 2010; Jao et al., 2015; Devos et al., 2019). In addition, many nursing home residents are not capable of shaping the sound environment to their needs due to diminished mental and physical capabilities (Boller et al., 2002).

For nursing staff, the nursing home is their workspace, and they tolerate different sounds and higher sound levels than nursing home residents. Although the nursing home is a home for the residents, the nursing staff has the biggest influence on the soundscapes in nursing homes. They shape the sound environment of their workspace, and are the main source of sound in nursing homes (Sloane et al., 2003). Nevertheless, staff in nursing homes has reported being irritable, and anxious, and having difficulties concentrating on their work due to poor soundscapes (McClaugherty et al., 2000). Despite these negative effects of poor soundscapes in nursing homes on residents and staff, studies about soundscape improvement in nursing homes are scarce (Janus et al., 2021).

Recent soundscape research in residential facilities has produced a smartphone application called Mobile Soundscape Appraisal and Recording Technology (MoSART; SoundAppraisal, Groningen, NL). It requires staff to record the sounds in their environment at random moments during the day and assess the soundscape quality as they perceive it. A pilot study among residents with profound intellectual multiple disabilities showed that raising soundscape awareness with the MoSART app decreased negative moods, problem behavior, and severity of problem behavior (van den Bosch et al., 2018a). Another pilot study was performed in nursing homes for residents with dementia. Although neither of the two participating venues had any formal soundscape knowledge, the nursing staff was found to be implicitly aware of the importance and role of sound in the care situation. The original application was then expanded to include ambassadors, i.e. specially trained staff members who ensure continued sound awareness in the nursing teams and facilitate improvements of the auditory environment. This elaborated intervention is called MoSART+ (van den Bosch and Andringa, 2018).

**Figure 1:**
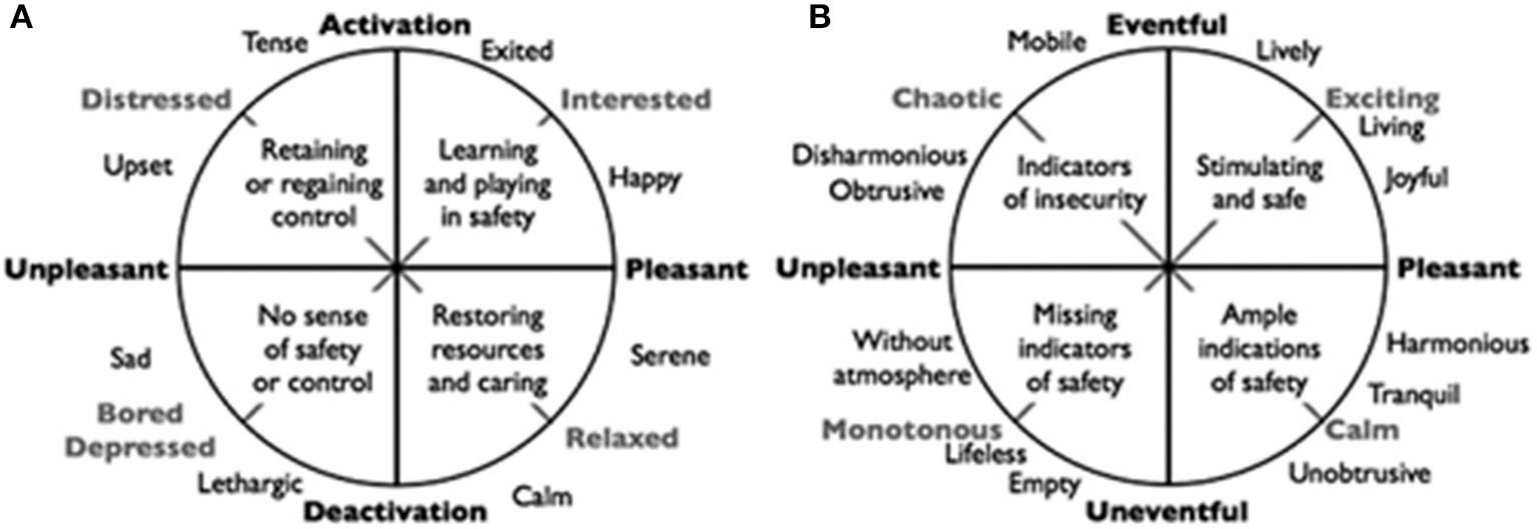
Core affect and appraisal of auditory environments (from Andringa and Lanser, 2013, CC-BY). **(A)** Core effects. **(B)** appraisal.

The MoSART+ intervention is based on the soundscape theory. This theory emphasizes that not the physical properties of sound (e.g., loudness), but the message conveyed by the sound and how the person in the environment perceives this message have the largest effect on wellbeing (Ising and Kruppa, 2004; International Organisation for Standardisation, 2014). This person-environment interaction is mediated by core-affect also often called mood (Russell, 2003). Axelsson et al. (2010) noticed that people describe the outdoor auditory environment usually in terms of pleasantness and eventfulness. Pleasantness indicates the extent to which an individual can flourish in a soundscape, whereas eventfulness reflects the investment needed to respond adequately to threats and opportunities (van den Bosch et al., 2018a). This shows that people describe their mood in a way that closely resembles the state of their surroundings. These deep evolutionary roots of core-affect and mood indicate that Axelsson's model is widely applicable to (outdoor and indoor) soundscapes. Theory development led to a classification of the environment in four categories: lively, calm, boring, and chaotic (Andringa and Lanser, 2013). A lively environment offers audible safety and multiple options that attract attention. A calm environment has many (often subtle) indicators or audible safety that allow relaxation and recovery of stress or challenges. A chaotic environment does not feel safe because it is difficult to interpret due to its complexity and the fast, cognitively intractable changes in sound. It is also associated with an increased risk of overstimulation. Lastly, boring soundscapes are environments devoid of indications of audible safety, typically because indicators of audible safety are masked, e.g., by machines like air conditioners. In this soundscape, the risk of understimulation is increased. The four categories are determined by four attributes (Figure 1) (Andringa and Lanser, 2013): pleasantness, eventfulness, complexity, and range of affordances. The affordance attribute indicates the extent to which the environment offers options for self-selected behavior. The complexity attribute indicates how difficult it is to choose appropriate behavior on basis of the environment. Based on these attributes, the categories chaotic and boring can be described as soundscapes that one wants to avoid. Calm and lively soundscapes are considered to be positive and favorable (van den Bosch et al., 2016).

After the successful pilot studies, the next step was to evaluate the effect of the MoSART+ intervention on problem behavior in residents with dementia. To that end, we set up a cluster-randomized controlled trial in five nursing homes. Intermediate outcomes were implemented micro-interventions and changes in the quality of the soundscapes. We report the effect that MoSART+ had on these outcomes in this article.

## Methods and Materials

### Design

**Figure 2:**
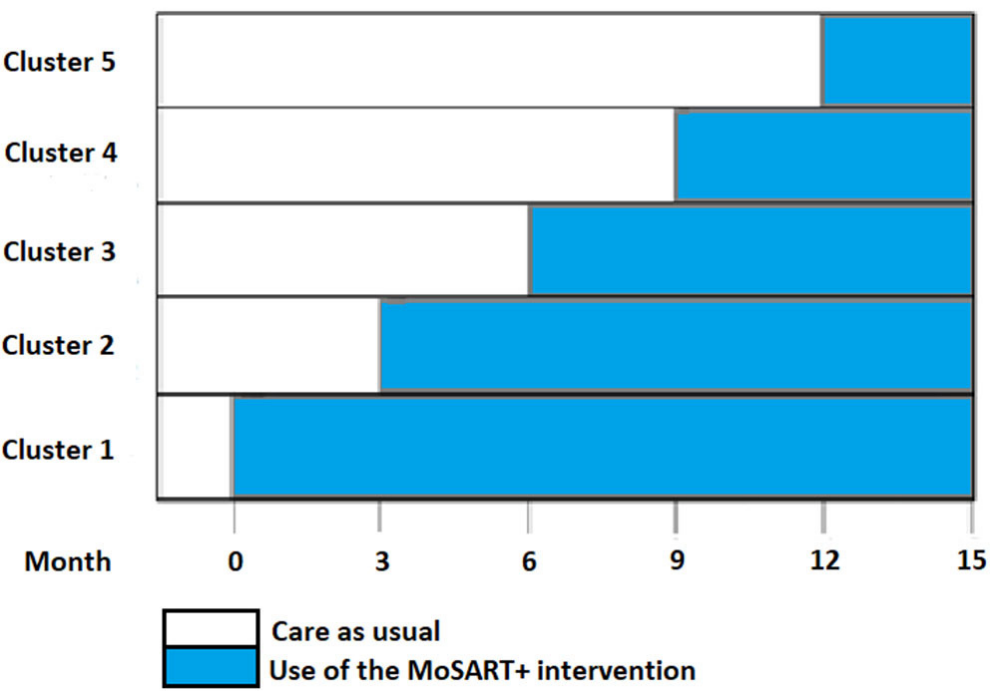
Stepped-wedge cluster design over 15 months.

We set out to perform a stepped wedge cluster-randomized trial in five nursing homes with each four or five dementia care units with a shared living room affiliated with the University Network of Elderly Care Organizations of the University Medical Center Groningen (UNO-UMCG). Every 3 months, one of five nursing homes switched from care-as-usual to the use of the MoSART+ intervention (Figure 2). The five clusters (one per nursing home) were randomized to one of five periods (steps). The allocation sequence was computer-generated prior the start of the trial and the allocations concealed from the nursing homes until the preparation for the intervention started in a home (e.g., ambassador selection). In the end, we implemented the MoSART+ intervention in four of the five nursing homes, because one organization suffered from staff shortage. The study protocol was approved by the Medical Ethics Review Committee of the University Medical Centre Groningen. A legal representative and, if possible, the resident provided informed consent.

### Study Population

For this article, the study population consisted of the staff that participated in the study. We aimed to include as many as possible staff members to obtain as many opinions and sound sensitivities as possible. This involved the nursing staff, nurses in training, psychologists, speech therapists, and activity therapists. An oral explanation about informed consent and participation in the study was given during the start meeting with the nursing staff. In addition, staff members signed an informed consent form, and the terms and conditions for use of the app the first time they opened it for an assessment. These terms and conditions again contained an informed consent. Individual members of the nursing staff that agreed to participate in the intervention gave their name and e-mail address to the ambassadors after the start meeting. With this information, the ambassadors were able to create individual private accounts for the nursing staff members in the app. Communal accounts were created when requested by the nursing home for use by flex workers and staff who did not want their private name and e-mail address to be used.

### Intervention

**Figure 3:**
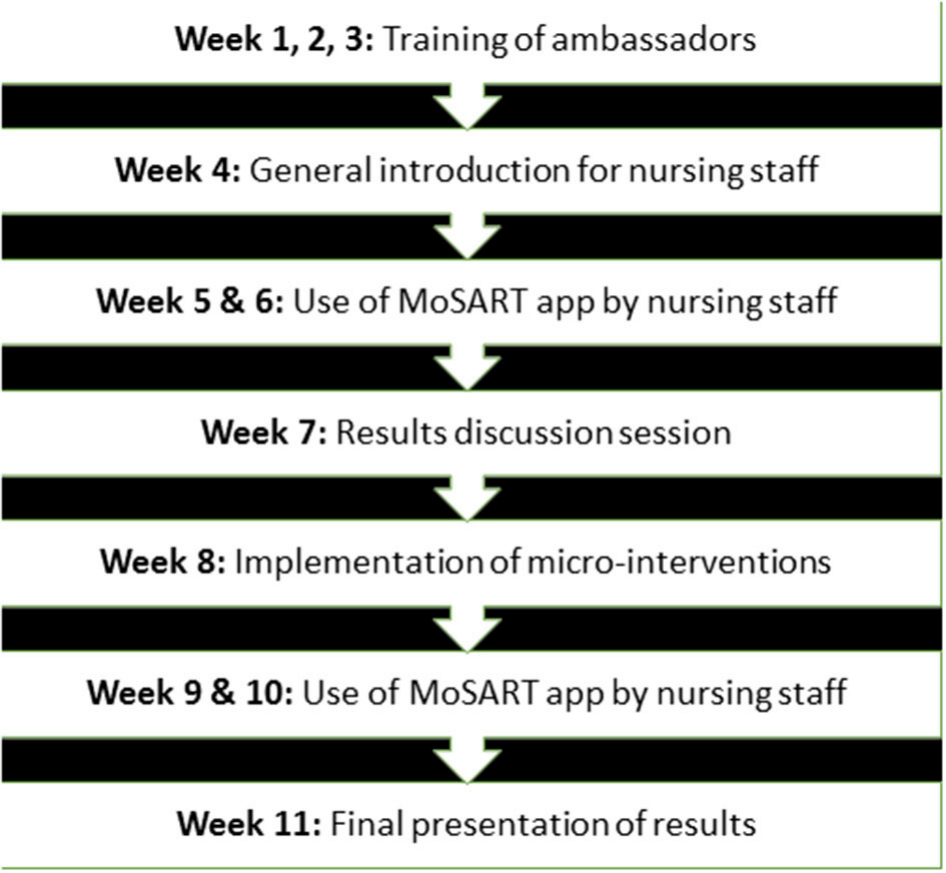
Chronological overview of the MoSART+ intervention by week.

The MoSART+ intervention consisted of four training sessions for ambassadors, the use of the MoSART smartphone app, meetings for the nursing staff, and micro-interventions (Figure 3). We started with three training sessions for ambassadors. In each nursing home, we selected two to four ambassadors. They were authoritative professionals with interest in or experience with the role of sound, and had to be familiar with the dynamics of the nursing team. The training sessions provided them with background knowledge on soundscape theory, the relationship between auditory environments and psychological wellbeing, and the implementation and use of the MoSART-app. The role of the ambassadors was to ensure sufficient participation, to answer questions about the procedure, and to facilitate a staff wide (informal) discussion about the role of sound in the nursing homes.

Next, an introduction meeting was organized for the nursing staff to provide them with information about sounds and the use of the app. The app was provided on a Xiaomi Mi A1 phone with phone operation system Android 8.0.without any apps other than MoSART. The app makes a 30–120 s audio recording while the nurse fills in a short questionnaire and appraises the auditory environment (see below for details). The nurses appraised the soundscape from their own perspective. Paying attention to the soundscape explicitly will create awareness about the auditory environment. To ensure privacy, the audio recordings were anonymized. Staff received three to four random cues per shift from the mobile phone to make a measurement.

The nursing staff was asked to use MoSART for 2 weeks. After these 2 weeks, the ambassadors and staff met to discuss the measurements. They also devised micro-interventions that could improve the soundscapes in the communal spaces, e.g., use of a tablecloth, and fixing a squeaky door. Ambassadors coordinated the execution of the micro-interventions during the next 2 weeks. After these 2 weeks, measurements with the MoSART app were resumed for another 2 weeks. The final meeting focused on discussing differences between the first and second set of measurements, and how consolidation of improvements could be realized. The MoSART+ intervention took about 10 weeks. The end goal was to empower the nursing staff to question and optimize the auditory environment.

### Outcomes

We collected the following outcomes related to the soundscapes: the room where the recording was made, sound sources, quality of the soundscape, and implemented micro-interventions. As mentioned above, the nursing staff used the MoSART app to assess the soundscapes. During each measurement, they answered questions about the room where the recording was made, sources of sound, and the quality of the sound environment in terms of the soundscape attributes, and graded the auditory environment. The room where the recording was made was rated in the categories: “living room/activity room,” “bedroom/ bathroom,” “kitchen,” “corridor,” “office,” and “garden/outdoors.” Sound sources were rated in the categories: “people,” “nature,” “traffic,” “music, TV and radio,” “machines and appliances,” and “other.” The soundscape attributes pleasantness, eventfulness, complexity, and range of affordances were rated on a scale of 0–100. Grades were given from 0 (very bad) to 10 (very good). At the end of the questionnaire, there was room for remarks. The app then presented the soundscape dimension that fitted the answers filled in, and the nursing staff had to indicate whether they agreed or not (van den Bosch, 2015). See a full description of the app and pictures in Supplementary Appendix.

The researchers noted the type and number of micro-interventions that were carried out.

### Data Analysis

First, we calculated the number of measurements and, the proportion of women among the staff and profession. Next, we calculated the percentage of measurements that were made in the communal rooms (living room/ activity room, kitchen, corridor, garden/outdoors) before and after implementation of the micro-interventions. Further, we calculated the percentage of different categories of sound sources before and after the implementation of the micro-interventions in the four nursing homes.

Then, we analyzed the quality of the soundscapes before and after the micro-interventions were implemented. We calculated the means with standard deviations and medians with interquartile range (IQR) of the scores on the soundscape attributes for all organizations together and each individually. We tested for differences between organizations with one-way ANOVA. Subsequently, we analyzed the pre-post changes in the soundscapes in the four nursing homes and per home with t-tests. The data was checked on skewness and outliers.

Finally, we calculated the proportions of the four soundscape dimensions, the distribution of the nurses' grades, and the means with a standard deviation of the grades for the four nursing homes in the periods before and after the micro-interventions were implemented. Changes in the percentage of soundscape dimensions and the proportions of grades ≥6 (“satisfactory”) were tested with a one-tailed pair-wise proportion Z-test. Again, data was checked on skewness and outliers.

All statistical tests used a significance level of 95%. Data was analyzed in Rstudio (R Studio Team, 2015). Micro-interventions were listed by category.

## Results

Staff in the four nursing homes made 1,882 measurements with 88 different MoSART accounts. Before micro-intervention were implemented, 1,195 measurements were made. The number of measurements per home varied between 115 and 512. Post micro-interventions, 687 measurements were made. The number per home varied between 236 and 790. 89% of the measurements were made in the communal spaces of the nursing homes (living-room, kitchen, garden, corridor) before and after implementation of the micro-interventions. Of the app users, 85% were female. Seven different professions contributed to the measurements: nurse assistant, nurse, psychologist, speech therapist, activity therapist, or nurse assistant in training.

### Sound Sources

**Figure 4:**
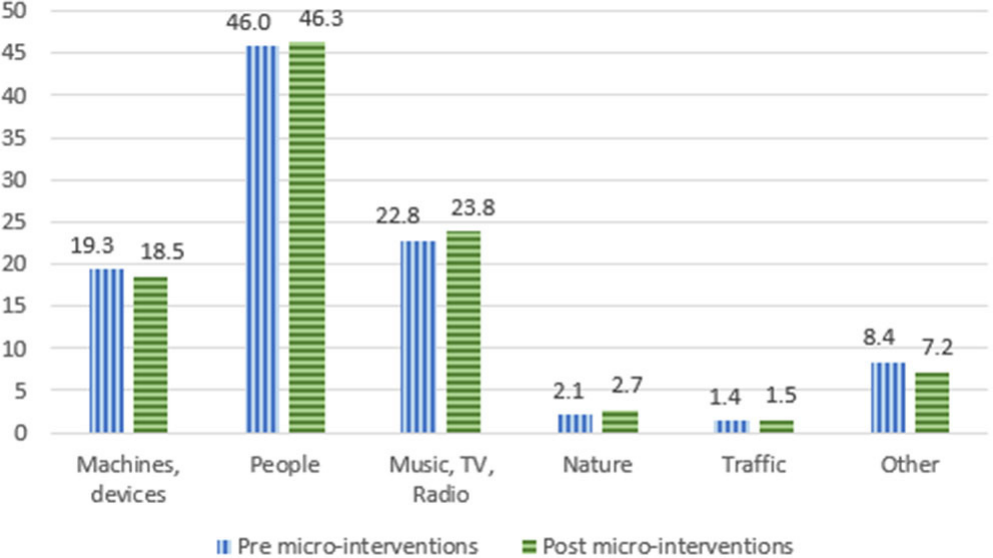
Sources of sounds pre- and post-implementation of micro-intervenntions (%).

The types of sound sources documented during the measurements is displayed in Figure 4. Before micro-interventions were implemented, the most frequent sound sources were “people” (46.0%). “Music, TV, and radio” (22.8%), as well as “machines and devices” (19.3%) were the second and third most common sound source in the measurements. The same sound sources were dominant after implementation of the micro-interventions. “People” being present in 46.3% of the measurements, “music, TV and radio” in 23.8%, and “machines and devices” in 18.5%.

### Quality of Soundscapes

#### Soundscape Attributes

Table 1 shows the measured soundscape attributes before the implementation of micro-interventions. In the four nursing homes, the soundscapes were mostly pleasant (median 69.0; IQR 49.0–83.5), of low complexity (32.0; 16.0–51.0), and uneventful (28.0; 13.0–53.0), and they gave moderate affordances (53.0; 36.0–70.5). One nursing home (number 3) scored higher on pleasantness and lower on complexity, eventfulness and affordances, compared to the other nursing homes.

**Table 1:** Soundscape attributes in participating nursing homes pre- and post-implementation of micro-interventions.

**Nursing home**	**Measurements, N**		Soundscape attribute*											
			**Unpleasant–pleasant**			**High complexity–low complexity**			**Few affordances–many affordances**			**Uneventful–eventful**		
			**M (SD)**	**Mdn (IQR)**	**MD (95% CI), *p***	**M (SD)**	**Mdn (IQR)**	**MD (95% CI), *p***	**M (SD)**	**Mdn (IQR)**	**MD (95% CI), *p***	**M (SD)**	**Mdn (IQR)**	**MD (95% CI), *p***
1	Pre	303	57.1 (27.7)	64.0 (39.0–77.0)	4.8 (54.9–59.6), <0.01	39.2 (25.9)	35.0 (20.0–56.0)	−4.1 (34.4–38.9), <0.01	53.8 (23.2)	56.0 (40.0–71.5)	−6.3 (49.2–53.4), <0.01	37.0 (27.5)	30 (16.0–57.5)	−8.7 (30.7–5.4), <0.01
	Post	132	61.9 (18.4)	65.5 (52.8–74.0)		35.1 (17.7)	32.0 (22.0–45.0)		47.5 (19.8)	49.5 (29.8–60.3)		28.3 (16.2)	27.0 (17.0–36.3)	
2	Pre	265	62.0 (24.0)	66.0 (47.0–80.0)	0.4 (58.6–63.0), <0.01	38.2 (24.4)	35.0 (18.0–55.0)	−0.3 (34.6–38.9), <0.01	50.3 (25.9)	52.0 (31.0–70.0)	−2.8 (45.5–50.2), <0.01	35.5 (25.9)	32.0 (14.0–54.0)	0.7 (32.0–36.8), <0.01
	Post	156	62.4 (21.1)	63.5 (49.0–76.0)		37.9 (19.7)	36.0 (24.0–52.0)		47.5 (21.4)	46.0 (34.8–62.3)		(36.2 23.1)	32.0 (23.0–48.0)	
3	Pre	115	69.8 (20.2)	71.0 (54.5–87.0)	−0.5 (65.5–70.6), <0.01	26.1 (19.3)	24.0 (10.0–38.0)	3.3 (23.8–28.9), <0.01	46.4 (22.1)	46.0 (35.0–58.0)	5.2 (44.6–50.4), <0.01	25.9 (21.7)	21.0 (9.0–39.0)	2.3 (22.8–28.4), <0.01
	Post	121	69.3 (19.5)	73.0 (54.0–84.0)		29.4 (20.4)	27.0 (12.0–44.0)		51.6 (23.0)	51.0 (39.0–68.0)		28.2 (22.1)	26.0 (10.0–44.0)	
4	Pre	512	67.9 (23.5)	74.0 (53.0–86.0)	3.2 (65.8–68.9), <0.01	33.6 (23.7)	30.5 (14.0–48.0)	−0.3 (30.5–33.8), <0.01	52.6 (25.8)	55.0 (36.0–70.5)	−0.6 (49.3–52.9), <0.01	33.7 (26.4)	28.0 (11.0–52.0)	1.3 (30.1–33.7), <0.01
	Post	278	70.2 (20.8)	73.0 (60.3–86.0)		33.3 (20.4)	31.5 (15.0–47.0)		52.0 (25.8)	51.5 (34.3–72.8)		32.4 (26.1)	25.0 (12.0–49.8)	
Total	Pre	1,195	64.0 (24.9)	69.0 (49.0–83.5)	2.7 (62.6–64.7), <0.01	35.3 (24.3)	32.0 (16.0–51.0)	−1.3 (32.4–34.5), <0.01	52.1 (24.9)	53.0 (36.0–70.5)	−2.0 (48.8–51.1), <0.01	34.2 (26.3)	28.0 (13.0–53.0)	−2.5 (30.8–33.1), <0.01
	Post	687	66.7 (20.5)	69.0 (54.0–81.0)		34.0 (20.7)	33.0 (18.0–47.0)		50.1 (23.5)	50.0 (35.0–67.0)		31.7 (23.3)	27.0 (14.0–46.5)	

*CI, confidence interval; IQR, interquartile range; M, mean; SD, standard deviation; Mdn, median; MD, differences in means between pre- and post-implementation of micro-interventions; p, p-value*;

*Attributes were scored from 0 to 100 (higher is better).

Table 1 also shows the soundscape attributes after implementation of the micro-interventions. The soundscapes were again mostly pleasant (median 69.0; IQR 54.0–81.0), of low complexity (33.0; 18.0–47.0), and uneventful (27.0; 14.0–46.5), and they gave moderate affordances (50.0; 35.0–67.0). Pleasantness and affordances statistically significantly differed between nursing homes (*p* < 0.05). No differences between homes were found for complexity and eventfulness.

The pre-post changes were small on average but consistent and statistically significant (*p* < 0.01) throughout the four organizations. They were larger within the individual homes. Eventfulness increased statistically significantly in two nursing homes and decreased in the two other nursing homes (*p* < 0.01).

#### Soundscape Dimensions

**Figure 5:**
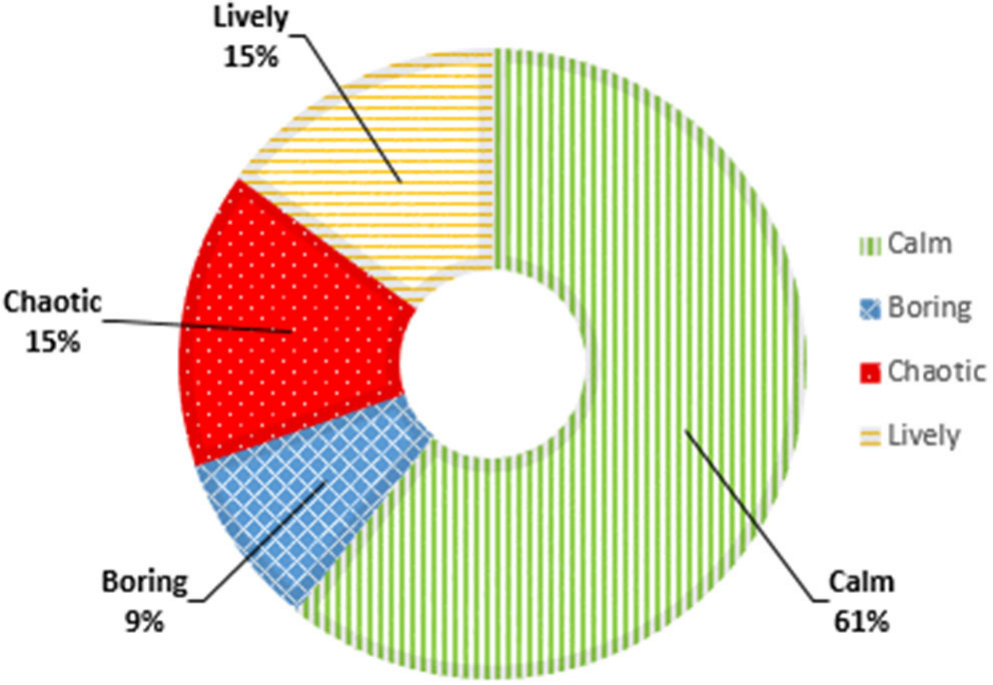
Soundscape dimensions pre-implementation of micro-interventions.

Figure 5 shows the soundscape dimensions before the implementation of micro-interventions. A calm soundscape was most prevalent in the nursing homes (61%; range 56–98%). In addition, 15% of the measurements indicated a lively soundscape, 15% a chaotic soundscape, and 9% a boring soundscape. In one nursing home, chaotic was the least common soundscape (8%), but in the other nursing homes boring was the least common soundscape (range 8–11%).

**Figure 6:**
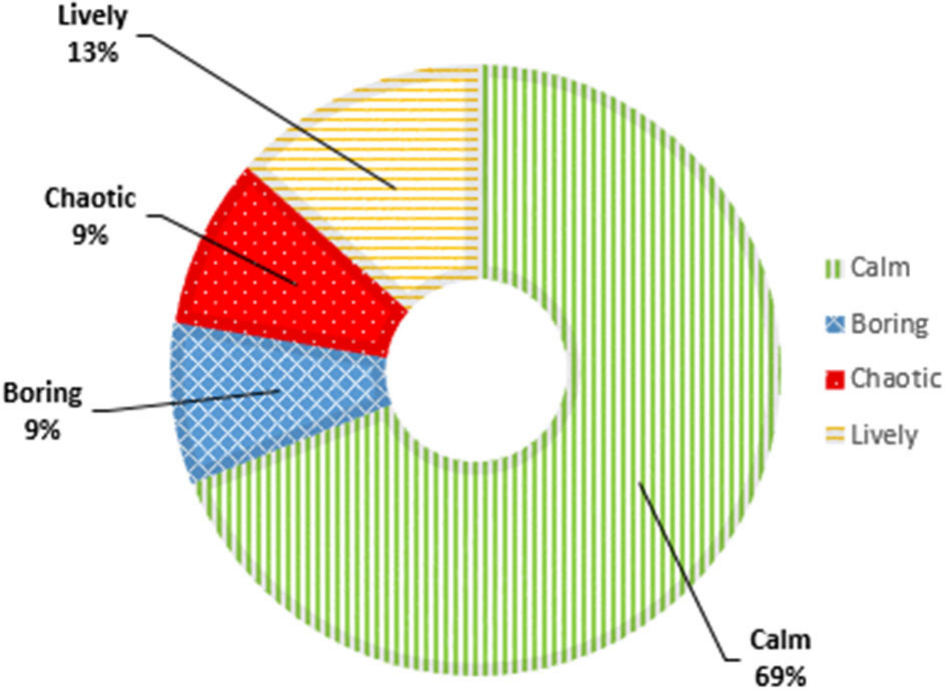
Soundscape dimensions post-implementation of micro-interventions.

Figure 6 shows the proportions of soundscape dimensions after implementation of MoSART+. The proportion of calm soundscapes increased from 61 to 69% (z = 12.7, *p* < 0.01), and the proportion of chaotic soundscapes decreased from 15 to 9% (z = 15.0, *p* < 0.01). The proportion of lively soundscapes decreased too (15–13%), but this change was not statically significant (z = 0.68, *p* = 0.79). The proportion of boring soundscapes did not change (9%).

#### Grades

**Figure 7:**
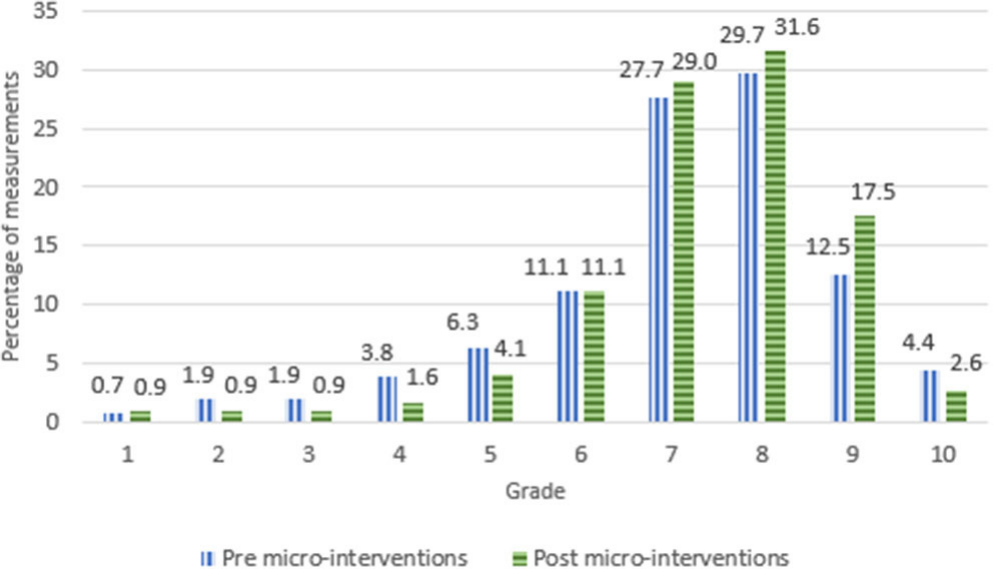
Grades given to the soundscape pre- and post-implementation of micro-interventions.

Figure 7 shows the distribution of the grades the nursing staff gave to the environment. Across all nursing homes, the environments were graded as satisfactory (≥6) in 1,021 measurements (85%) before the micro-interventions were implemented. The percentages of satisfactory grades varied between 81 and 88%, and the mean score between 6.7 and 7.4.

After implementation of micro-interventions, the percentage of satisfactory grades was 91%. It varied between 88 and 95%, and the mean score between 7.4 and 8.0. Hence, the proportion of satisfactory grades (≥6) increased from 85 to 91% after implementation of the micro-interventions, and this change was statistically significant (z = 15.3, *p* < 0.01).

### Micro-interventions

Table 2 shows the micro-interventions carried out in the nursing homes. The interventions were unique to each nursing home and depended on the building itself, and the specific needs of the nursing staff and the residents. Nevertheless, some micro-interventions were carried out by multiple nursing homes. These were: acoustical panels to reduce noise, use of (new) more silent electrical medicine grinders, repairment of the (loud) entrance door, reduction of noise of bed rails/pump, closing doors when the (dish) washing machine is used, and announcing the use of a household appliance. Two nursing homes also informed all colleagues of the micro-interventions, either on a blackboard in the nurses' office, or by email. Three of the nursing homes set up a protocol for family and colleagues on how to enter the ward. All in all, the micro-interventions were mainly focused on removing disturbing sounds, and not on adding positive sounds to the environment.

**Table 2:** Micro-interventions carried out during the study in the nursing homes.

**Category of intervention**	**Interventions carried out (number of nursing homes)**
Soft interventions	Brake on kitchen drawer (1), pads on kitchen door (1), wrap blender in towel (1), acoustic panels (2), nature sounds in corridor of the ward by a whistling decorative bird statue with movement sensor (1), nature sounds at the beginning of the day by using youtube and a speaker(1)
Machine	New electrical medicine grinder (2), specific notifications per ward on beepers (1), new water boiler (1), unpacking dishwasher quietly (2), set radio on tuner radio suited for residents (2), new food steamer (1), have maintenance team look at entrance door (2), have maintenance team fix squeaky office chairs (1), contact municipality for reduce sound of ringing bells when a nearby bridge opened (1), reduce noise of bed rails/pump (2), reduce noise while cooking (1), use mop instead of sweeper on wards (1)
Protocols	Closing doors when washing machine is used (2), rules about entering the ward for family and colleagues (3), announcing the use of a household appliance (2), mention all interventions at the day start board (1), email all interventions to colleagues (1), transfer of shift outside of ward or in empty bedroom (1), discuss welcome or goodbye on ward with family (1), pick up incontinence material outside of ward (1)
Other	Ask if children in adjacent daycare can go outside at a later time (1), discuss extra staff to adequately guide residents with agitation (1), create a room for shouting residents with little sensory input to recover from overstimulation for a short period of time (1), camera's in bedrooms for nightshift instead of nightly check-ups in person to prevent disruption (1)

## Discussion

In our study in four nursing homes, the main sound sources before and after implementation of micro-interventions were people, music, TV, radio machines, and devices. After implementation of micro-interventions, the proportion of calm soundscapes increased at the expense of the proportion of chaotic and lively soundscapes. No difference in the proportion of boring soundscapes occurred. The grades given to the auditory environment increased after implementing the micro-interventions. The micro-interventions focused on removing or reducing disturbing sounds and were unique for every nursing home.

The measurements showed that the most dominant sound sources were people, music, TV, radio, machines, and devices (together 88%). Nature and traffic contributed the least to the auditory environments in the participating nursing homes. Our findings align with previous research results. A recent review also showed that human vocal sounds and electronic sounds were most dominant in nursing homes (Janus et al., 2021). The human vocal sounds were related to the nursing staff (29% up to 34%) and resident (35%), and electronic sounds to equipment, environmental (telephones ringing/ doors slamming), cleaning equipment and alarms (Janus et al., 2021). In addition, an online survey showed that the human vocal sounds and the electronic sounds were most dominant and noticeable (Aletta et al., 2018).

The mean scores on the soundscape attributes suggest that the soundscapes became more pleasant, less complex, and less eventful and gave fewer affordances. However, the median and interquartile range revealed that unpleasant measurements became less unpleasant instead of more pleasant, and boring measurements became less boring instead of livelier. The median also indicated a less complex environment and an environment that offered fewer affordances. Hence, it seems that MoSART made the soundscapes less unpleasant, less complex, less eventful, and offering fewer affordances.

The improvement of the soundscapes was confirmed by the decrease in negative soundscapes. After the implementation of the micro-interventions, the chaotic soundscapes statistically significantly decreased from 15 to 9%. Although the absolute difference of 6% seems small, the relative reduction of 40% in a negative soundscape that may greatly affect nurse home residents through overstimulation, may be clinically relevant. Reducing overstimulation is especially important for residents with dementia, as their ability to filter out unwanted stimuli is reduced (Fleming and Purandare, 2010). In the end, the environment has become less complicated to understand and less unpleasant than before.

We expected that MoSART+ would also reduce boring soundscapes, and reduce the risk of understimulation in the residents. However, the proportion of boring soundscapes was not affected. This is in line with our observation that nursing staff concentrated on reducing negative and disturbing sounds instead of adding pleasant sounds to the environment. Nevertheless, staff in the nursing home with the highest soundscape quality at the start also tried to embed positive sounds in the environment through the introduction of natural sounds, e.g., adding nature sound in the corridor or at the start of the day. It is possible that nursing staff first has to reduce disturbing sounds in the environment before being able to add positive soundscape to the environment. Perhaps, observing and reducing boring soundscapes also requires a higher level of awareness than observing and reducing disturbing sounds.

The positive effect of some micro-interventions on the soundscape may not be constant, e.g., announcing the use an appliance or closing doors may be applied variably by the nurses. In contrast, modifications of the physical environment, such as pads on kitchen doors or nature sounds in the corridor, provide a stable improvement of the soundscape. Therefore, the success of a micro-intervention implementation depends in some cases on the nursing home as also noted by Thomas et al. (2020). Which makes sense, since sounds are produced by particular situations and activities and not by continual stationary processes. Therefore, we suggest to firstly implement modifications of the physical environment and additionally micro-interventions that are less constant.

Most micro-interventions were aimed at reducing machine noise. However, the sound sources did not change substantially after the implementation of the micro-interventions. Nevertheless, the soundscapes were less chaotic and the nursing staff were more satisfied with the sound environment as reflected by their grades. This would indicate that awareness of the mechanical sound sources increased, and that staff started to use devices in more appropriate ways and at more appropriate times. Such factors may be more important for improving auditory environments than changing the sound sources itself (Devos et al., 2019).

One nursing home showed different results compared to the other three nursing homes. In this home, MoSART+ was implemented during the summer vacation period. Many health care professionals were on holiday, and hence, flex workers were dominant. These flex workers were often only present for a day or couple of days, which was disruptive for the implementation process, and led to the inclusion of many different opinions, sensitivities to sound, and background knowledge in the role of sound. These workers had not received proper training about the intervention either. This experience taught us that implementation of the MoSART+ can best be done under stable circumstances.

During the intervention periods, we observed that sound sensitivity and awareness differed between staff. At the start, some staff members mentioned that they were never annoyed by noise, while others were. Some persons became more aware in a shorter period than others, and some were already very aware of the influence of sounds on the residents. The nursing staff reported that making the measurements and taking time to listen to the environment, made them notice more (annoying) sounds than before. In addition, as a result of discussing the measurements and different opinions, they became more aware of differences in their sensitivity, and the influence of sounds on mood and behavior of the residents, e.g., in one nursing home, a door produced a clicking noise and a hard bang when closing. Staff observed that the sounds startled many of the residents. It turned out that they reminded the residents about shootings in the second world war. Some members of the nursing staff also reported that it had become impossible to turn their “noise filter” on again, and they kept hearing all the sounds in the environment. These experiences and the study results show that MoSART+ enhanced the sound awareness among staff, which in turn enabled them to implement practical micro-interventions to improve the environment.

### Strengths and Weaknesses

To our knowledge, no other study about improving soundscapes in nursing homes through raising awareness has been performed. We consider this a strength of our study. The flexibility and freedom in choosing ambassadors and micro-interventions that fitted the nursing home best is a strength of the intervention. This flexibility makes it possible to adapt the auditory environment to different residents and situations. Especially new residents in nursing homes demand a lot of flexibility and possibilities to discover what fits this resident and the group best since the in-group dynamics also change every time a new resident moves in.

Another strength of the intervention was the training of the ambassadors in the nursing homes. Together with the nursing home staff, we selected two to four ambassadors per nursing home with different educational backgrounds and sonic preferences. As a result, different perspectives on soundscapes were highlighted during the meetings. Further, the ambassadors encouraged the nursing staff to perform assessments with the MoSART+ app, and they were able to ensure that the micro-interventions fitted in with the rules and procedures of the nursing homes, and the wishes of the nursing staff and residents. The presence of ambassadors thus enhanced sound awareness among the nursing staff and effective implementation of the micro-interventions. Additionally, through teaching the ambassadors, knowledge is retained in the organizations after the intervention stopped, and the durability and effects of the intervention were secured.

A weakness of the study is the difference in the number of measurements before and after the implementation of the micro-interventions. Based on the discussions in the meetings, we assume that this difference can be attributed to increased awareness. However, the question remains whether staff ignored cues from the app at random or not. It is possible that the nursing staff focused more on positive soundscapes post-implementation of micro-interventions. In this case, the reduction in the proportion of chaotic soundscapes could have been lower than was found in our study. On the other hand, if the nursing staff focused more on negative soundscapes and sounds, as was the case with the micro-interventions, the real decrease in chaotic soundscapes was larger than we observed.

A weakness of the intervention was that special phones had to be used for the MoSART+ application instead of the phones that staff already carried on them. The extra phone was easily forgotten. On top of that, the special phones depended on WiFi connectivity in the nursing homes, which sometimes functioned poorly. As a result, notifications were not always received, and measurements were not synchronized to the database. In fact, one nursing home suffered from a WiFi failure for a week during one of the post micro-intervention measurement periods. Therefore, fewer measurements were received and the time planning had to be readjusted.

### Implications for Future Research

Soundscape improvement by raising awareness through the MoSART+ intervention positively affected the soundscapes and the nursing teams' satisfaction with them. As this is the first study of its kind, our results need to be reproduced. We encourage future research to investigate ways to add positive sounds to the personal spaces of residents based on their personal preferences (Devos et al., 2018, 2019). Further, in some situations, residents with dementia are capable of indicating which sounds are disturbing or not. Therefore, we suggest that the nursing staff probe the viewpoint of the residents more structurally as part of the measurements.to the intervention. This would improve the interaction between the residents and their environment even further.

We also recommend restricting study participation to organizations that are stable enough to carry out a long-term intervention, and to periods outside the summer holidays. This will prevent dropout of organizations and will lead to more reliable and valid results. We also recommend to incorporate an app as much as possible in the work routine of the nursing staff, for example by installing it on the phones that they already use. This will make it easier for them to collect data.

## Conclusion

We conducted a trial in which the MoSART+ soundscape awareness intervention resulted in an improved auditory environment. Nursing staff was empowered to evaluate and adapt the soundscapes. Given that soundscapes are often poor in nursing homes and negative effects have been shown on residents and staff, nursing homes should now consider improving the auditory environment of private and communal spaces with MoSART+.

## Data Availability Statement

For verification purposes the following data will be made available on request: various versions of processed data, documentation about the data, documentation about the research process, raw data, and syntaxes. The audio recordings are not readily available to ensure the privacy of the participants. For follow-up research, data will be available in consultation with Prof. S. U. Zuidema (s.u.zuidema@umcg.nl) and Project Leader H. J. Luijendijk (h.j.luijendijk@umcg.nl). Requests to access the datasets should be directed to s.u.zuidema@umcg.nl.

## Ethics Statement

The studies involving human participants were reviewed and approved by Medical Ethics Review Committee of the University Medical Centre Groningen. The patients/participants provided their written informed consent to participate in this study.

## Author Contributions

JK wrote the paper, analyzed the data, collected the data, and carried out the intervention. SJ formulated the research questions, designed the study, and assisted with writing the article and collecting the data. KV and SZ formulated the research questions and designed the study. HL formulated the research questions, designed the study, supervised the conduct of the study, provided feedback during data analysis, and assisted with writing the article. TA formulated the research question, designed the study, provided feedback during data analysis, assisted with writing the article, and carried out the intervention. All authors approved the final version.

## Funding

This work was supported by ZonMw (733050833). ZonMw is a Dutch non-profit organization that finances health research and healthcare innovation. ZonMw was not involved in the collection, analysis, and interpretation of data, the writing of the report, or in the decision to submit the article for publication.

## Conflict of Interest

TA was employed by SoundAppraisal BV. The remaining authors declare that the research was conducted in the absence of any commercial or financial relationships that could be construed as a potential conflict of interest.

## Publisher's Note

All claims expressed in this article are solely those of the authors and do not necessarily represent those of their affiliated organizations, or those of the publisher, the editors and the reviewers. Any product that may be evaluated in this article, or claim that may be made by its manufacturer, is not guaranteed or endorsed by the publisher.
