# Supplementary material for: Soundscape Optimization in Nursing Homes Through Raising Awareness in Nursing Staff With MoSART+
Source: Front Psychol. 2022 Jun 1;13:871647. doi: 10.3389/fpsyg.2022.871647 (PMC9199855; doi:10.3389/fpsyg.2022.871647)
Supplement: Supplementary file 1 [file Data_Sheet_1.docx]

**Appendix I. The MoSART app**


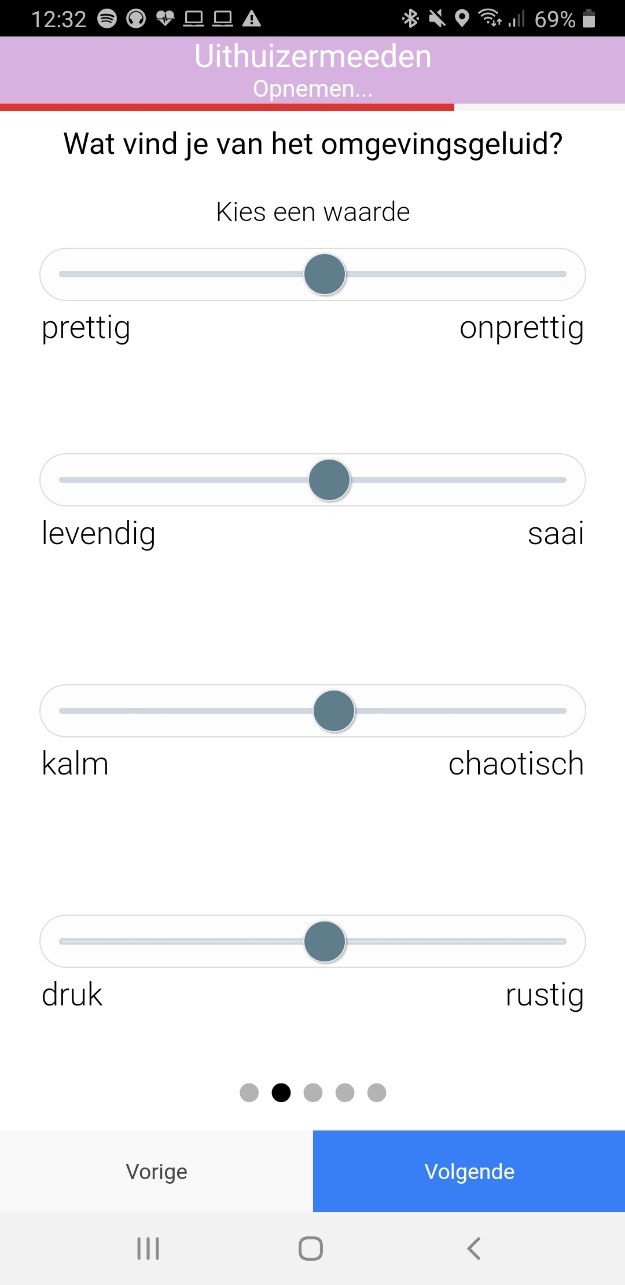

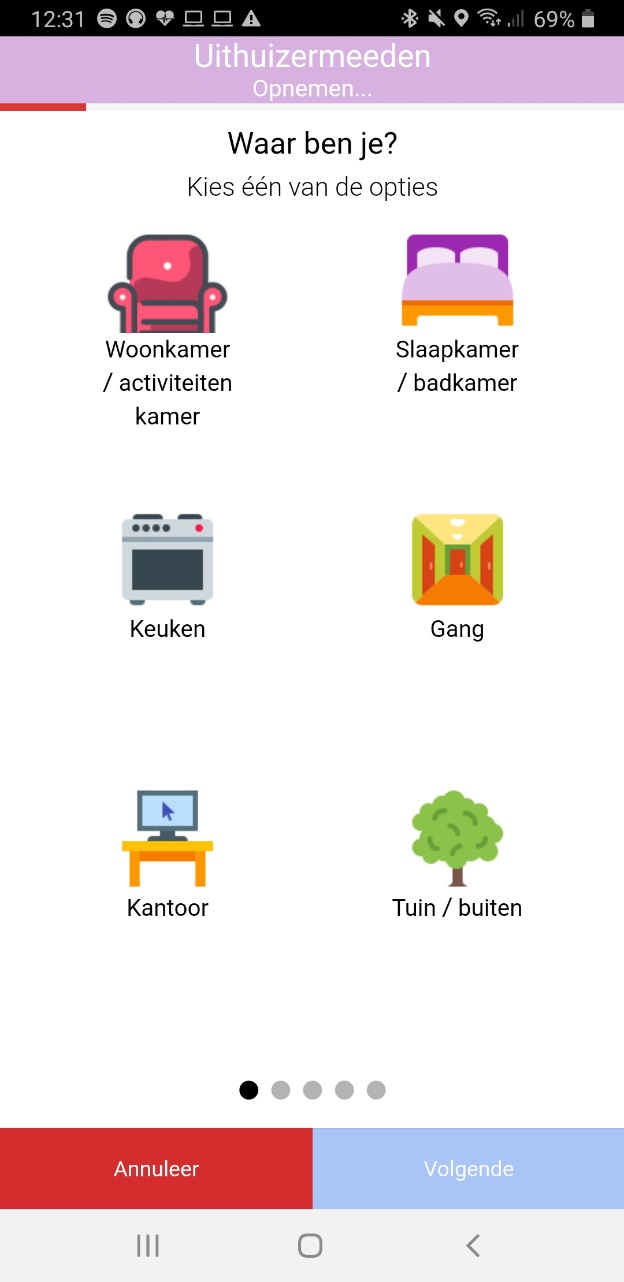
This appendix shows the Dutch MoSART app in screenshots below. The screenshots are presented in chronological order, as presented in the app. For privacy reasons of the nursing home, the screenshots are cropped at the upper side of the app. Translation of the questions to English can be found after the screenshots. (Images from Soundappraisal. Reproduced with permission.)


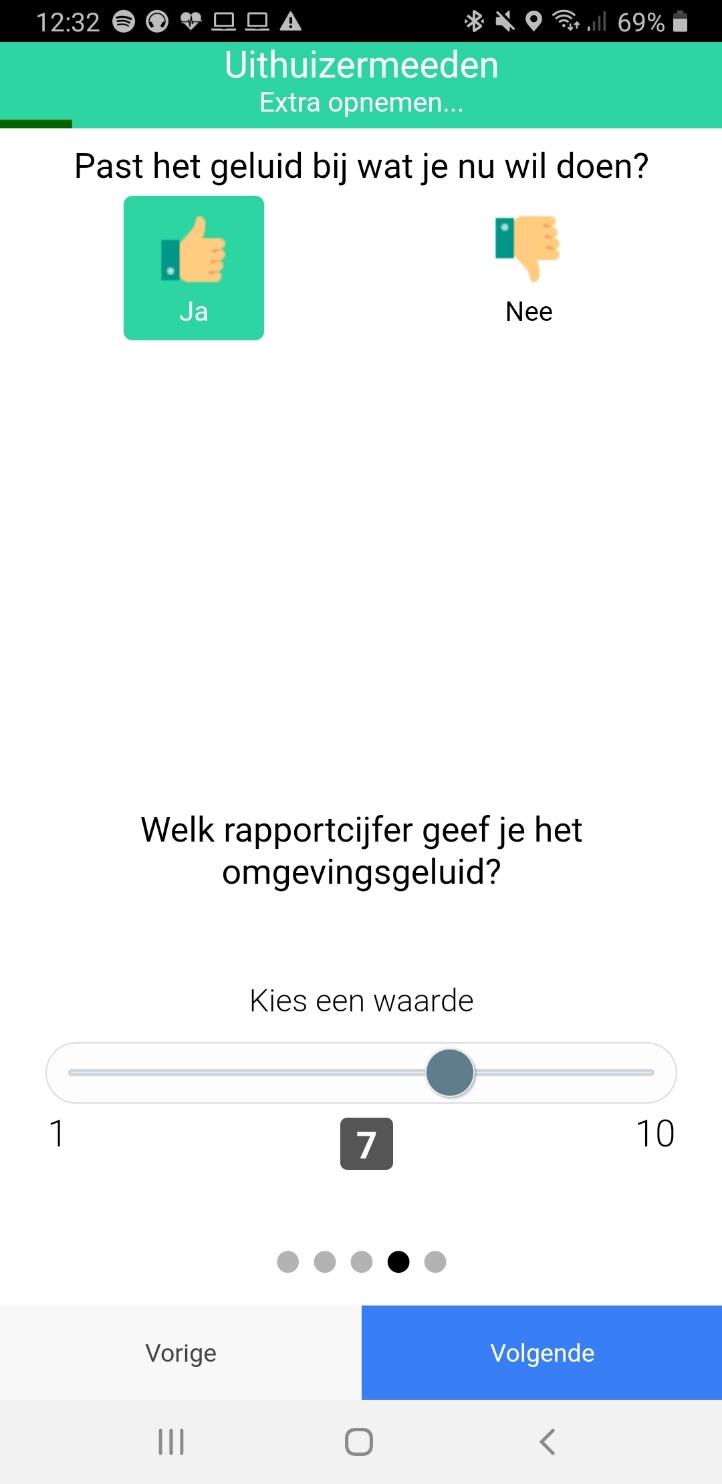

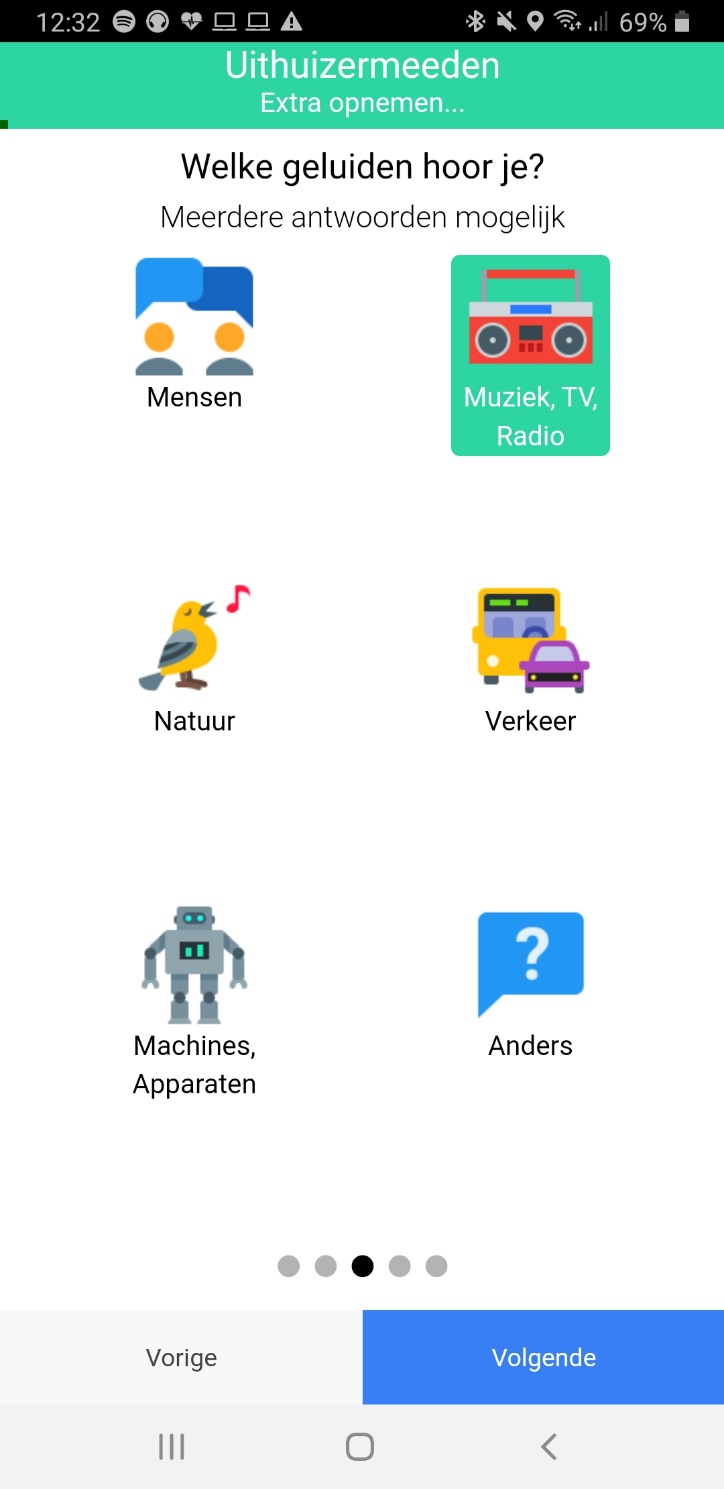


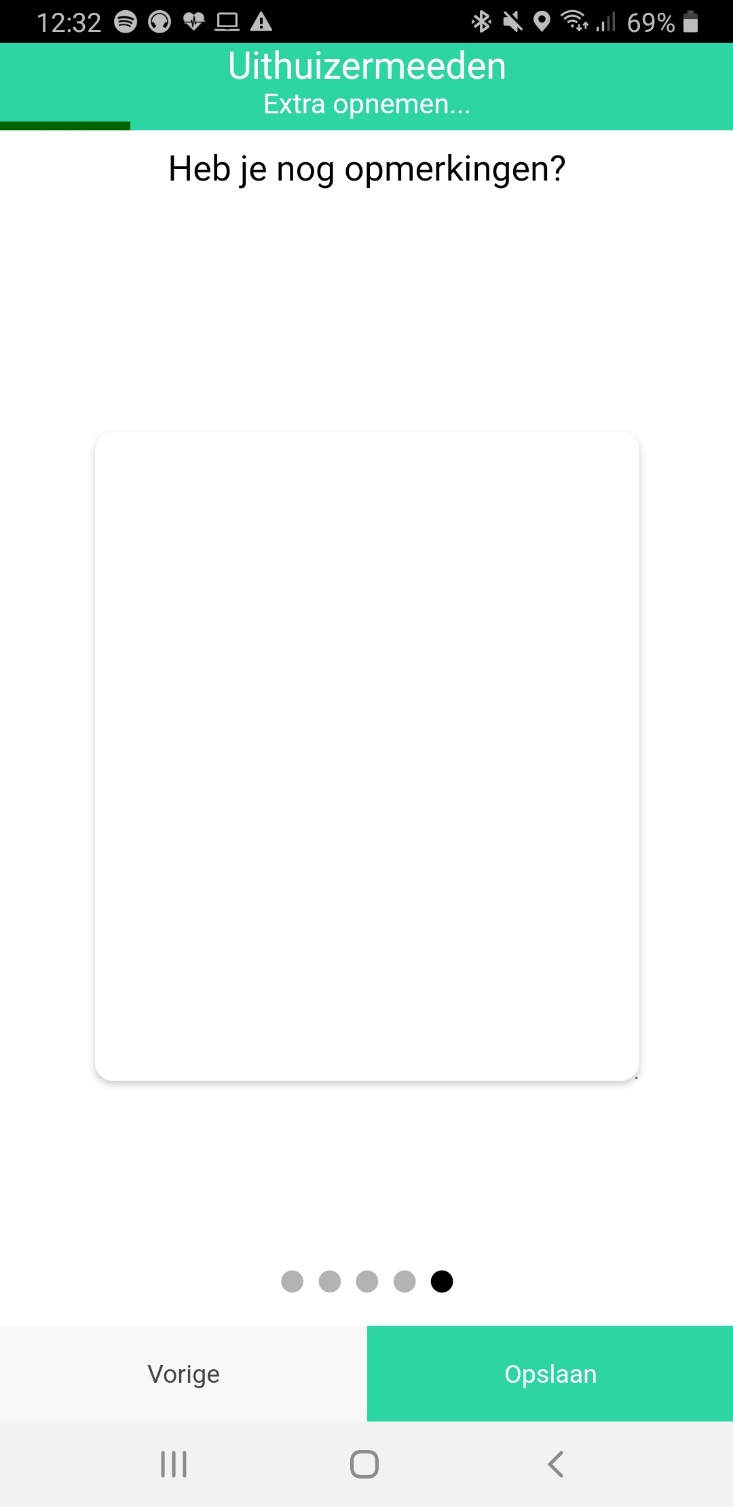


**Translation of the questions:**

Question 1: Where are you?

Answers (select one):

- Living room / activity room
- Bedroom / bathroom
- Kitchen
- Corridor
- Office
- Garden / outdoors

Question 2: What do you think of the sonic environment?

Answers (scale of 0 to 100):

- Pleasant – Unpleasant
- Lively – Boring
- Calm – Chaotic
- Eventful – Uneventful

Question 3: What sounds do you hear?

Answers (Multiple answers possible):

- People
- Music, TV, Radio
- Nature
- Traffic
- Machines, devices
- Other

Question 4: Does the sound match with what you want to do now?

Answers (select one):

- Yes
- No

Question 5: How would you grade the sonic environment?

Answer on a scale of 1 (very bad) to 10 (very good).

Question 6: Do you have any remarks?

Answer can be typed in the box.
